# Associations of continuous anionic gap detection with the mortality in critically ill patients receiving renal replacement therapy

**DOI:** 10.1007/s11255-023-03583-4

**Published:** 2023-04-07

**Authors:** Yiling Zhai, Changjun Luo, Tao Zhou, Guangzhi Zeng, Qiongyan Huang, Jun Li

**Affiliations:** 1 Department of Emergency Medicine, Affiliated Liutie Central Hospital of Guangxi Medical University, Liuzhou, 545007 Guangxi China; 2 Department of Cardiovascular Medicine, Affiliated Liutie Central Hospital of Guangxi Medical University, Liuzhou, 545007 Guangxi China; 3 Department of Critical Care Medicine, Affiliated Liutie Central Hospital of Guangxi Medical University, Liuzhou, 545007 Guangxi China; 4 Department of Coronary Heart Disease Intensive Care Unit, Affiliated Liutie Central Hospital of Guangxi Medical University, Liuzhou, 545007 Guangxi China; 5 Liuzhou Key Laboratory of Molecular Diagnosis, Guangxi Key Laboratory of Molecular Diagnosis and Application, Affiliated Liutie Central Hospital of Guangxi Medical University, Liuzhou, 545007 Guangxi China

**Keywords:** Renal replacement therapy, Serum anion gap, Dialysis, ∆AG

## Abstract

**Purpose:**

To investigate the associations of anion gap (AG) levels before and 1-day after hemodialysis as well as anion gap changes with the mortality in critically ill patients receiving renal replacement therapy (RRT).

**Methods:**

Totally, 637 patients from MIMIC-III were included in this cohort study. The associations between AG (T0), AG (T1), or ∆AG [AG (T0) − AG (T1)], and the risk of 30-day or 1-year mortality were examined by Cox restricted cubic spline regression models. Univariate and multivariate Cox proportional-hazards model was applied to assess the associations between AG (T0), AG (T1), ∆AG with 30-day and 1-year mortality, respectively.

**Results:**

The median follow-up time was 18.60 (8.53, 38.16) days and 263 (41.3%) patients were survived. There was a linear relationship between AG (T0), AG (T1) or ∆AG and the risk of 30-day or 1-year mortality, respectively. The risk of 30-day mortality was higher in AG (T0) > 21 group (HR = 1.723, 95% CI 1.263–2.350), and AG (T1) > 22.3 group (HR = 2.011, 95% CI 1.417–2.853), while lower in AG > 0 group (HR = 0.664, 95% CI 0.486–0.907). The risk of 1-year mortality was increased in AG (T0) > 21 group (HR = 1.666, 95% CI 1.310–2.119), and AG (T1) > 22.3 group (HR = 1.546, 95% CI 1.159–2.064), while decreased in AG > 0 group (HR = 0.765, 95% CI 0.596–0.981). Patients with AG (T0) ≤ 21 had higher 30-day and 1-year survival probability than those with AG (T0) > 21.

**Conclusion:**

AG before and after dialysis as well as the changes of AG were important factors associated with the risk of 30-day and 1-year mortality in critically ill patients receiving RRT.

**Supplementary Information:**

The online version contains supplementary material available at 10.1007/s11255-023-03583-4.

## Introduction

Renal replacement therapy (RRT) is required in patients with critical illness in intensive care units (ICUs) who suffer from a great burden of comorbidities and being unable to tolerate the hemodynamic shifts of conventional hemodialysis [1]. RRT provides organ support in critically ill patients with kidney failure for the management of fluid when the kidneys are unable to meet the metabolic demand of the critically ill disease state [2, 3]. Although the application of RRT increased the survival benefits for many patients in ICUs, the mortality of patient require RRT was as high as 60% in some studies [4–6]. To deep investigate more related prognostic biomarkers associated with critically ill patients receiving RRT might help the clinicians to improve the outcomes of these patients.

The serum anion gap (AG) is a mathematical derivation parameter obtained based on the difference in serum cation and anion concentrations [7]. AG reflects the unmeasured concentration of anion, which is considered to be a biomarker assessing the acid–base disorders and helping identify different forms of metabolic acidosis [8]. Along with differential diagnosis of acid–base, this value can be used as a biomarker for predicting prognosis in patients with chronic kidney disease [9]. Previously, the increase of AG in patients with moderate chronic kidney disease was reported to be associated with elevated risk for progression to end-stage renal disease [10]. Cheng et al. revealed that serum AG was an effective predictor for all-cause mortality in critically ill patients with acute kidney injury [7]. A recent study found that there is a “J-shaped” association between AG before dialysis and mortality in elderly patients over 75 years old who receive hemodialysis, indicating too high or too low AG might lead to higher risk of death [11]. The AG is a time-varying factor associated with the renal outcomes [12], but current studies were mainly explored the AG at some time point. Monitoring the AG and its changes at different time points may be instructive for providing appropriate interventions for critically ill patients to improve their outcomes. There was evidence indicated that the acid–base balance before hemodialysis was significantly different from that after hemodialysis in stable hemodialysis patients [13]. The pre and post- hemodialysis serum AG was also different [14]. Whether the changes of AG levels before and after hemodialysis were associated with the mortality in critically ill patients receiving RRT needs exploration.

In this study, the purpose was to investigate the associations of AG levels before and 1-day after hemodialysis as well as anion gap changes at the two time points with the mortality in critically ill patients receiving RRT using the data from the Medical Information Mart for Intensive Care-III (MIMIC-III) database. Subgroup analysis was performed based on the age, the estimated glomerular filtration rate (eGFR), the Elixhauser Comorbidity Index (ECI).

## Methods

### Study design and population

Totally, 687 patients undergoing RRT with the measurement of AG calculation related biomarkers from MIMIC-III between 2001–2012 were involved in this cohort study. MIMIC-III included the health data of over 40,000 patients who were admitted to the intensive care unit (ICU) of Beth Israel Deaconess Medical Center [15]. The information of patients including demographics, vital signs, laboratory findings, imaging reports, organ failure scores, severity of illness scores, comorbidities, diagnosis, treatment, length of stay in hospital, and survival data were collected [16]. Patients ≥ 18 years who received RRT for ≥ 2 days in ICU from the database with measurement of anion gap before and 1-day after RRT were included in our study. The requirement of ethical approval for this was waived by the Institutional Review Board of Affiliated Liutie Central Hospital of Guangxi Medical University, because the data was accessed from MIMIC-III (a publicly available database). All individuals provided informed consent before participating in the study. In our study, patients aged < 18 years, and those with RRT treatment < 2 days were excluded. Finally, 637 participants were included.

### Main variables

Age (years), gender, ethnicity (White, Black, Asian, Hispanic/Latino, or others), systolic blood pressure (SBP, mmHg), diastolic blood pressure (DBP, mmHg), respiratory rate (time/min), heart rate (time/min), temperature (℃), oxygen saturation (SpO_2_, %), fraction of inspired oxygen (FiO_2_, %), white blood cell (WBC, K/UL), neutrophil (%), lymphocytes (K/UL), platelets (PLT, K/UL), hemoglobin (g/dL), red cell distribution width (RDW), total bilirubin (TBIL, μmol/L), albumin (g/dL), blood urea nitrogen (BUN, mg/dL), creatinine (mg/dL), eGFR (mL/min), glucose (mg/dL), lactate (mmol/L), pH, base excess, AG (T0), AG (T1),, urine output, fluid input (mL), length of stay (LOS, day), oxygen therapy, mechanical ventilation, vasopressor, ECI, Glasgow coma scale (GCS), the Simplified Acute Physiology Score II (SAPSII), Sequential Organ Failure Assessment (SOFA).

AG (T0) referred to AG levels before dialysis and AG (T1) indicated AG levels 1-day after dialysis. Values of AG were calculated using the following equation: AG (serum sodium [mmol/L] + serum potassium [mmol/L]) − (serum chloride [mmol/L] + serum bicarbonate [mmol/L]) + 2.5 × (4 − observed albumin [g/dL]) [17]. ∆AG = AG (T0) − AG (T1). The participants were stratified into two groups according to the cut-point of AG (T0), AG (T1), and ∆AG, respectively including AG (T0) ≤ 21 mmol/L and AG (T0) > 21 mmol/L, AG (T1) ≤ 22.3 mmol/L and AG (T1) > 22.3 mmol/L, ∆AG ≤ 0 and ∆AG > 0. AG (T0) ≤ 21 mmol/L, AG (T1) ≤ 22.3, mmol/L ∆AG ≤ 0 were set as the reference groups respectively. The X-tile software was used for cut-point selection.

### Outcome variables

Survival information (including survival outcome and time of death) was obtained from the Social Security Death Index records. The follow-up endpoints were all-cause mortality at 30 days and 1 year after ICU admission. The median follow-up after ICU admission to discharge or death was 18.60 (8.53, 38.16) days.

### Statistical analysis

Continuous variables were presented as mean and standard deviation (mean ± SD) or median and interquartile range [IQR], and categorical variables were displayed as number (percentages) [*n* (%)]. Differences in baseline variables between groups stratified by the patients who were survival and dead were compared via Mann–Whitney or *t* tests for continuous variables, and the Chi-squared test or Fisher’s exact test for dichotomous variables. The associations between AG (T0), AG (T1), or ∆AG, and the risk of 30-day or 1-year mortality were examined by Cox restricted cubic spline regression models. Univariate and multivariate Cox proportional-hazards model was applied to assess the association between AG (T0), AG (T1), ∆AG with 30-day and 1-year mortality, respectively. Survival analysis was performed using standardized Kaplan Meier curves. Subgroup analysis were stratified for age (< 65 years or ≥ 65 years), eGFR (< 15 or ≥ 15), ECI (< 25 or ≥ 25). Multivariate model adjusted for age, respiratory rate, heart rate, SBP, DBP, respiratory failure, SPO2, PLT, RDW, TBIL, eGFR, urine output, oxygen therapy, mechanical ventilation, vasopressor ECI, SAPSII, SOFA. A two-sided *P* value of < 0.05 was considered statistically significant. All analyses were performed using R version 4.1.2 (R Foundation for Statistical Computing, Austria, Vienna).

## Results

### The baseline characteristics of all participants

**Fig. 1:**
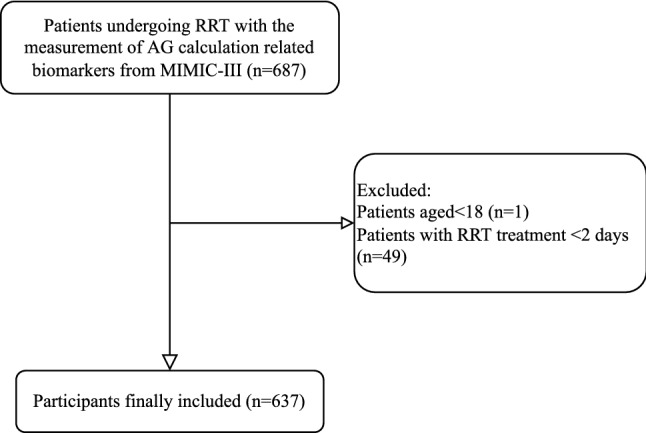
The screen process of the participants

Overall, 687 patients were involved in this study. We excluded 1 patient aged < 18 years, and 49 patients with RRT treatment < 2 days, and 637 participants were finally included. The screen process of the participants was shown in Fig. 1. Among them, 219 participants died within 30 days, and 335 participants died within 1 year after ICU admission. All participants were divided into the survival group (*n* = 263) and death group (*n* = 374) based on the overall survival data from the database. As shown in Table 1, the median age in the death group was older than the survivor group (66.42 years vs 59.04 years). The mean SBP (117.18 mmHg vs 126.97 mmHg) and DBP (62.86 mmHg vs 67.32 mmHg) in the death group were lower than the survival group. The median creatinine in the death group was lower than the survival group (2.40 mg/dL vs 3.10 mg/dL). The median eGFR in the death group was higher than the survival group (29.70 mL/min vs 23.36 mL/min). The mean AG (T0) (22.52 vs 21.15) and AG (T1) (20.43 vs 18.95) in the death group was higher than the survival group. The mean ECI in the death group was higher than the survival group (32.18 vs 23.89).

**Table 1:** The characteristic of the critically ill patients receiving renal replacement therapy

Variables	Total (*n* = 637)	Survival (*n* = 263)	Death (*n* = 374)	*P*
Age (median [IQR])	63.45 (52.25, 74.65)	59.04 (47.89, 69.11)	66.42 (56.12, 77.82)	< 0.001
Gender, *n* (%)				
Female	256 (40.2)	109 (41.4)	147 (39.3)	0.645
Male	381 (59.8)	154 (58.6)	227 (60.7)	
Ethnicity, *n* (%)				
White	411 (64.5)	161 (61.2)	250 (66.8)	0.062
Black	84 (13.2)	45 (17.1)	39 (10.4)	
Asian	16 (2.5)	8 (3.0)	8 (2.1)	
Hispanic/Latino	28 (4.4)	11 (4.2)	17 (4.5)	
Other	21 (3.3)	12 (4.6)	9 (2.4)	
COPD, *n* (%)				
No	573 (90.0)	242 (92.0)	331 (88.5)	0.187
Yes	64 (10.0)	21 (8.0)	43 (11.5)	
Respiratory failure, *n* (%)				
No	274 (43.0)	138 (52.5)	136 (36.4)	< 0.001
Yes	363 (57.0)	125 (47.5)	238 (63.6)	
Lung cancer, *n* (%)				
No	632 (99.2)	262 (99.6)	370 (98.9)	0.607
Yes	5 (0.8)	1 (0.4)	4 (1.1)	
Liver cirrhosis, *n* (%)				
No	498 (78.2)	225 (85.6)	273 (73.0)	< 0.001
Yes	139 (21.8)	38 (14.4)	101 (27.0)	
Renal failure, *n* (%)				
No	107 (16.8)	66 (25.1)	41 (11.0)	< 0.001
Yes	530 (83.2)	197 (74.9)	333 (89.0)	
Heart failure congestive, *n* (%)				
No	388 (60.9)	174 (66.2)	214 (57.2)	0.028
Yes	249 (39.1)	89 (33.8)	160 (42.8)	
AF, *n* (%)				
No	391 (61.4)	184 (70.0)	207 (55.3)	< 0.001
Yes	246 (38.6)	79 (30.0)	167 (44.7)	
Heart disease, *n* (%)				
No	524 (82.3)	225 (85.6)	299 (79.9)	0.086
Yes	113 (17.7)	38 (14.4)	75 (20.1)	
Hypertension, *n* (%)				
No	218 (34.2)	89 (33.8)	129 (34.5)	0.932
Yes	419 (65.8)	174 (66.2)	245 (65.5)	
Diabetes mellitus, *n* (%)				
No	441 (69.2)	177 (67.3)	264 (70.6)	0.425
Yes	196 (30.8)	86 (32.7)	110 (29.4)	
Hyperlipidemia, *n* (%)				
No	405 (63.6)	162 (61.6)	243 (65.0)	0.431
Yes	232 (36.4)	101 (38.4)	131 (35.0)	
Malignant cancer, *n* (%)				
No	521 (81.8)	226 (85.9)	295 (78.9)	0.03
Yes	116 (18.2)	37 (14.1)	79 (21.1)	
SBP (mean ± SD)	121.22 ± 27.97	126.97 ± 27.71	117.18 ± 27.47	< 0.001
DBP (mean ± SD)	64.70 ± 19.65	67.32 ± 21.06	62.86 ± 18.40	0.005
Respiratory rate (mean ± SD)	20.19 ± 6.52	19.54 ± 6.56	20.65 ± 6.46	0.034
Heart rate (mean ± SD)	93.12 ± 20.59	93.96 ± 21.24	92.53 ± 20.13	0.388
Temperature (mean ± SD)	36.49 ± 1.10	36.62 ± 1.06	36.41 ± 1.11	0.017
SpO_2_ (median [IQR])	98.00 (94.00, 100.00)	98.00 (95.00, 100.00)	98.00 (94.00, 100.00)	0.025
FiO_2_ (median [IQR])	88.00 (50.00, 100.00)	84.00 (50.00, 100.00)	88.00 (50.00, 100.00)	0.526
WBC (K/UL) (median [IQR])	10.20 (7.10, 15.30)	10.00 (7.65, 15.05)	10.50 (6.55, 15.50)	0.813
Neutrophil (%) (median [IQR])	82.00 (73.00, 88.00)	82.00 (73.00, 88.00)	82.05 (72.40, 88.00)	0.682
Lymphocytes (K/UL) (median [IQR])	9.60 (5.40, 15.20)	9.90 (5.25, 15.71)	9.50 (5.50, 14.47)	0.494
PLT, K/UL (median [IQR])	186.00 (119.00, 260.00)	208.00 (144.00, 275.00)	174.50 (109.00, 247.00)	< 0.001
Hemoglobin (g/dl) (mean (SD))	10.69 (2.32)	10.90 (2.56)	10.55 (2.13)	0.057
RDW (mean ± SD)	16.17 ± 2.34	15.64 ± 2.29	16.54 ± 2.30	< 0.001
TBIL (μmol/L) (median [IQR])	0.80 (0.40, 2.10)	0.70 (0.40, 1.58)	0.90 (0.41, 2.48)	0.033
Albumin (g/dL) (mean ± SD)	3.06 ± 0.70	3.16 ± 0.71	2.99 ± 0.69	0.002
BUN (mg/dl) (median [IQR])	40.00 (23.00, 65.00)	40.00 (23.00, 61.00)	41.00 (23.25, 67.00)	0.716
Creatinine (mg/dl) (median [IQR])	2.60 (1.40, 4.80)	3.10 (1.50, 5.90)	2.40 (1.40, 4.18)	< 0.001
eGFR (mL/min) (median [IQR])	28.37 (15.73, 52.69)	23.36 (12.16, 51.44)	29.70 (17.77, 52.98)	0.005
Glucose (mg/dl) (median [IQR])	128.00 (101.00, 175.00)	131.00 (102.00, 182.50)	127.00 (101.00, 168.75)	0.133
Lactate (mmol/L) (median [IQR])	1.90 (1.30, 3.10)	1.88 (1.28, 3.00)	1.95 (1.40, 3.20)	0.159
pH (median [IQR])	7.35 (7.26, 7.42)	7.34 (7.26, 7.42)	7.35 (7.26, 7.43)	0.366
Base excess (median [IQR])	− 3.00 (− 8.00, 1.00)	− 3.00 (− 8.00, 1.00)	− 3.00 (− 8.00, 0.75)	0.962
AG (T0) (mean ± SD)	21.97 ± 5.75	21.15 ± 5.49	22.52 ± 5.86	0.006
AG (T1) (mean ± SD)	19.79 ± 4.50	18.95 ± 3.77	20.43 ± 4.91	< 0.001
∆AG (mean ± SD))	1.95 ± 5.14)	2.39 ± 5.67)	1.62 ± 4.68)	0.075
Urine output (mL) (median [IQR])	208.00 (56.00, 641.00)	350.00 (90.50, 879.50)	134.00 (40.25, 449.25)	< 0.001
Fluid input (median [IQR])	578.00 (0.00, 1500.00]	500.00 (0.00, 1387.50)	600.00 (0.00, 1587.50)	0.459
LOS (day) (median [IQR])	8.55 (3.94, 16.01)	8.62 (3.88, 15.88)	8.26 (3.98, 16.96)	0.924
Oxygen therapy (%)	91 (14.3)	17 (6.5)	74 (19.8)	< 0.001
	546 (85.7)	246 (93.5)	300 (80.2)	
Mechanical ventilation (%)	137 (21.5)	68 (25.9)	69 (18.4)	0.032
	500 (78.5)	195 (74.1)	305 (81.6)	
Vasopressor (%)	425 (66.7)	210 (79.8)	215 (57.5)	< 0.001
	212 (33.3)	53 (20.2)	159 (42.5)	
ECI (mean ± SD)	28.76 ± 14.13	23.89 ± 13.84	32.18 ± 13.33	< 0.001
GCS (median [IQR])	15.00 (13.00, 15.00)	15.00 (14.00, 15.00)	15.00 (13.00, 15.00)	0.271
SAPS-II (mean ± SD)	51.96 ± 15.06	48.14 ± 14.42	54.65 ± 14.93	< 0.001
SOFA (median [IQR])	10.00 (7.00, 14.00)	10.00 (7.00, 13.00)	11.00 (8.00, 14.00)	0.008

*SBP* systolic blood pressure, *DBP* diastolic blood pressure, *SpO*_*2*_ oxygen saturation, *FiO*_*2*_ fraction of inspired oxygen, *WBC* white blood cell, *RDW* red cell distribution width, *TBIL* total bilirubin, *BUN* blood urea nitrogen, *ECI* Elixhauser comorbidity index, *GCS* Glasgow coma scale, *SAPS* Simplified Acute Physiology Score, *SOFA* Sequential Organ Failure Assessment, *eGFR* estimated glomerular filtration rate, *AG* anion gap

### The associations of AG (T0), AG (T1), and ∆AG with the 30-day mortality or 1-year mortality in patients

**Fig. 2:**
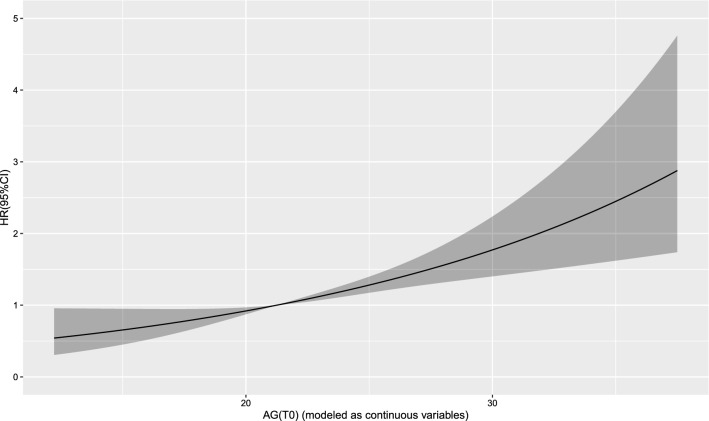
The Cox regression model with a restricted cubic spline showing a linear relationship between AG (T0) and the risk of 30-day mortality in the patients

**Fig. 3:**
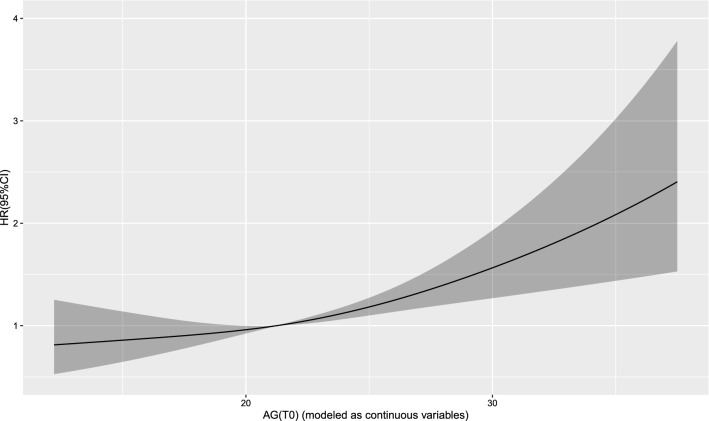
The Cox regression model with a restricted cubic spline showing a linear relationship between AG (T0) and the risk of 1-year mortality in the patients

**Fig. 4:**
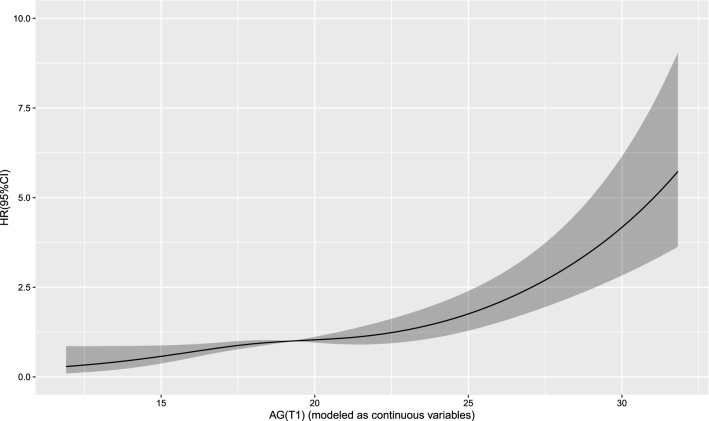
The Cox regression model with a restricted cubic spline showing a linear relationship between AG (T1) and the risk of 30-day mortality in the patients

**Fig. 5:**
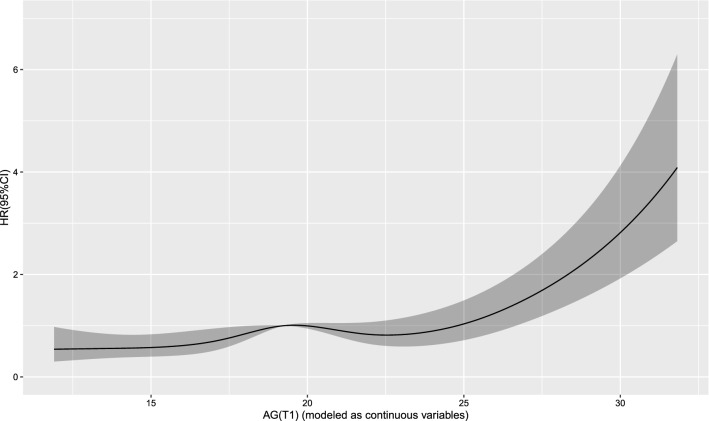
The Cox regression model with a restricted cubic spline showing a linear relationship between AG (T1) and the risk of 1-year mortality in the patients

**Fig. 6:**
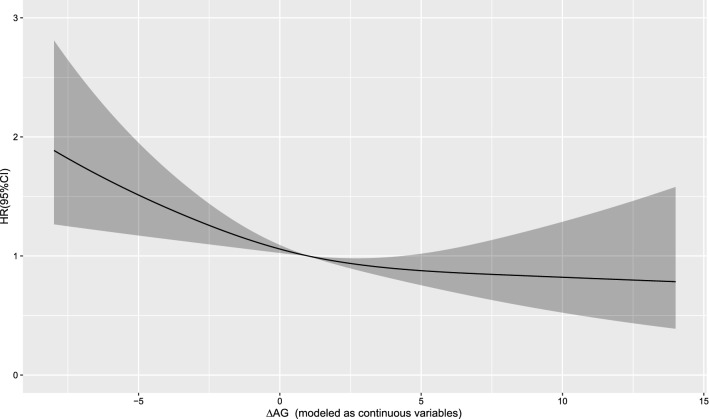
The Cox regression model with a restricted cubic spline showing a linear relationship between ∆AG and the risk of 30-day mortality in the patients

**Fig. 7:**
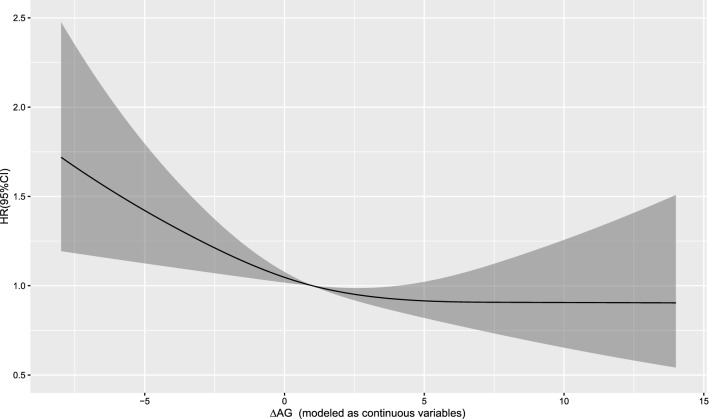
The Cox regression model with a restricted cubic spline showing a linear relationship between ∆AG and the risk of 1-year mortality in the patients

The Cox regression model with a restricted cubic spline showed a linear relationship between AG (T0) and the risk of 30-day mortality (Fig. 2) as well as the risk of 1-year mortality (Fig. 3) in the patients. A linear relationship between AG (T1) and the risk of 30-day mortality (Fig. 4) as well as the risk of 1-year mortality (Fig. 5) in the patients were observed in the Cox regression model with a restricted cubic spline. The Cox regression model with a restricted cubic spline showed a linear relationship between ∆AG and the risk of 30-day mortality (Fig. 6) as well as the risk of 1-year mortality in the patients (Fig. 7).

Table 2 shows the results of the Cox proportional-hazards model. After adjustment for the confounding factors including age, respiratory rate, temperature, SpO_2_, PLT, RDW, TBIL, eGFR, urine output, oxygen therapy, mechanical ventilation, vasopressor, ECI, SAPSII, SOFA, the risk of 30-day mortality was higher in AG (T0) > 21 group (HR = 1.723, 95% CI 1.263–2.350), and AG (T1) > 22.3 group (HR = 2.011, 95% CI: 1.417–2.853), while lower in AG > 0 group (HR = 0.664, 95% CI 0.486–0.907). After adjusting for age, respiratory rate, SBP, DBP, respiratory failure, temperature, SpO_2_, PLT, RDW, TBIL, eGFR, urine output, oxygen therapy, mechanical ventilation, vasopressor, ECI, SAPSII, SOFA and AG (T0), the risk of 1-year mortality was increased in AG (T0) > 21 group (HR = 1.666, 95% CI 1.310–2.119), and AG (T1) > 22.3 group (HR = 1.546, 95% CI 1.159–2.064), while decreased in AG > 0 group (HR = 0.765, 95% CI 0.596–0.981).

**Table 2:** Associations of AG (T0), AG (T1), and ∆AG with the 30-day mortality or 1-year mortality in patients

	30-Day mortality				1-Year mortality			
	Univariate model		Multivariate model^a^		Univariate model		Multivariate model^a^	
	HR (95% CI)	*P*	HR (95% CI)	*P*	HR (95% CI)	*P*	HR (95% CI)	*P*
AG (T0)								
≤ 21	Ref		Ref		Ref		Ref	
> 21	1.874 (1.410, 2.491)	< 0.001	1.723 (1.263, 2.350)	< 0.001	1.552 (1.238, 1.947)	< 0.001	1.666 (1.310, 2.119)	< 0.001
AG (T1)^b^								
≤ 22.3	Ref		Ref		Ref		Ref	
> 22.3	2.483 (1.829, 3.371)	< 0.001	2.011 (1.417, 2.853)	< 0.001	1.703 (1.318, 2.201)	< 0.001	1.546 (1.159, 2.064)	0.016
∆AG								
≤ 0	Ref		Ref		Ref		Ref	
> 0	0.581 (0.432, 0.78)	< 0.001	0.664 (0.486, 0.907)	0.011	0.678 (0.536, 0.857)	0.001	0.765 (0.596, 0.981)	0.045

*AG* anion gap

^a^Multivariate model adjusted for age, respiratory rate, heart rate, SBP, DBP, respiratory failure, SPO2, PLT, RDW, TBIL, eGFR, urine output, oxygen therapy, mechanical ventilation, vasopressor ECI, SAPSII, SOFA

^b^When analyzing AG (T1), multivariate model additionally adjusted for AG (T0) as a continuous variable

### The cumulative survival probability of 30-day mortality and 1-year mortality in patients

The cumulative survival probability of 30-day mortality among participants as stratified by AG (T0) levels was shown in Supplementary Fig. 1, which revealed that patients with AG (T0) ≤ 21 had higher 30-day survival probability than those with AG (T0) > 21. The cumulative survival probability of 1-year mortality among participants with AG (T0) ≤ 21 was higher than those with AG (T0) > 21 (Supplementary Fig. 2). The cumulative survival probability of 30-day mortality (Supplementary Fig. 3) and 1-year mortality (Supplementary Fig. 4) among participants with AG (T1) ≤ 22.3 were higher than those with AG (T1) > 22.3. The cumulative survival probability of 30-day mortality (Supplementary Fig. 5) and 1-year mortality (Supplementary Fig. 6) among participants with ∆AG > 0 levels were higher than those with ∆AG ≤ 0.

### Subgroup analysis of the associations of AG (T0), AG (T1), and ∆AG with the 30-day mortality or 1-year mortality in patients

In terms of different age groups, the risk of 30-day mortality was increased in patients aged ≥ 65 years with AG (T0) > 21 (HR = 2.111, 95% CI 1.356–3.286) and patients < 65 years with AG (T1) > 22.3 (HR = 2.558, 95% CI 1.552–4.215) after multivariate adjustment for confounders. In patients with different eGFR, we found that the risk of 30-day mortality was elevated in patients with AG (T0) > 21 in eGFR ≥ 15 group (HR = 2.061, 95% CI 1.485–2.861) and AG (T1) > 22.3 in eGFR < 15 group (HR = 3.757, 95% CI 1.060–13.318). The risk of 30-day mortality was decreased in patients with ∆AG > 0 in eGFR < 15 group (HR = 0.211, 95% CI 0.062–0.719). As for patients with different ECI levels, the increased risk of 30-day mortality was observed in those with AG (T0) > 21 in ECI ≥ 25 group (HR = 1.621, 95% CI 1.142–2.303) as well as AG (T1) > 22.3 in both ECI < 25 group (HR = 4.441, 95% CI 1.617–12.041) and ECI ≥ 25 group (HR = 1.641, 95% CI 1.097–2.454). The increased risk of 30-day mortality was found in patients with ∆AG > 0 in ECI < 25 group (HR = 0.485, 95% CI 0.247–0.951) and ECI ≥ 25 group (HR = 0.678, 95% CI 0.473–0.973) (Table 3).

**Table 3:** Subgroup analysis of the associations of AG (T0), AG (T1), and ∆AG with the 30-day mortality in patients

	Age				eGFR				ECI			
	< 65		≥ 65		< 15		≥ 15		< 25	≥ 25		
	HR (95% CI)	*P*	HR (95% CI)	*P*	HR (95% CI)	*P*	HR (95% CI)	*P*	HR (95% CI)	*P*	HR (95% CI)	*P*
AG (T0)												
≤ 21	Ref		Ref		Ref		Ref		Ref		Ref	
> 21	1.399 (0.901, 2.172)	0.135	2.111 (1.356, 3.286)	< 0.001	0.308 (0.073, 1.295)	0.108	2.061 (1.485, 2.861)	< 0.001	1.343 (0.637, 2.824)	0.437	1.621 (1.142, 2.303)	0.007
AG (T1)^a^												
≤ 22.3	Ref		Ref		Ref		Ref		Ref		Ref	
> 22.3	2.558 (1.552, 4.215)	< 0.001	1.405 (0.782, 2.524)	0.256	3.757 (1.060, 13.318)	0.040	1.463 (0.983, 2.175)	0.061	4.441 (1.617, 12.041)	0.004	1.641 (1.097, 2.454)	0.016
∆AG												
≤ 0	Ref		Ref		Ref		Ref		Ref		Ref	
> 0	0.639 (0.402, 1.016)	0.058	0.665 (0.422, 1.047)	0.078	0.211 (0.062, 0.719)	0.129	0.709 (0.510,0.987)	0.042	0.485 (0.247, 0.951)	0.035	0.678 (0.473, 0.973)	0.035

Multivariate model adjusted for age, respiratory rate, heart rate, SBP, DBP, respiratory failure, SPO_2_, PLT, RDW, TBIL, eGFR, urine output, oxygen therapy, mechanical ventilation, vasopressor ECI, SAPSII, SOFA

*AG* anion gap, *ECI* Elixhauser comorbidity index, *eGFR* estimated glomerular filtration rate

^a^When analyzing AG (T1), multivariate model additionally adjusted for AG (T0) as a continuous variable

As shown in Table 4, after multivariate adjustment for confounders, there was a higher risk of 1-year mortality among both age < 65 years group (HR = 1.819, 95% CI 1.261–2.622) and age ≥ 65 years group (HR = 1.579, 95% CI 1.1122–2.222), eGFR ≥ 15 group (HR = 1.851, 95% CI 1.428–2.398), and both ECI < 25 group (HR = 1.466, 95% CI 0.857–2.510) and ECI ≥ 25 group (HR = 1.476, 95% CI 1.115–1.954) in patients with AG (T0) > 21. A higher risk of 1-year mortality was observed in aged < 65 years group (HR = 1.992, 95% CI 1.308–3.032) and ECI < 25 group (HR = 2.547, 95% CI 1.178–5.747) in patients AG (T1) > 22.3. There was a lower risk of 1-year mortality among subjects ECI < 25 group in patients with ∆AG > 0 (HR = 0.582, 95% CI 0.347–0.978).

**Table 4:** Subgroup analysis of the associations of AG (T0), AG (T1), and ∆AG with 1-year mortality in patients

	Age				eGFR				ECI			
	< 65		≥ 65		< 15		≥ 15		< 25		≥ 25	
	HR (95% CI)	*P*	HR (95% CI)	*P*	HR (95% CI)	*P*	HR (95% CI)	*P*	HR (95% CI)	*P*	HR (95% CI)	*P*
AG (T0)												
≤ 21	Ref		Ref		Ref		Ref		Ref		Ref	
> 21	1.819 (1.261, 2.622)	0.001	1.579 (1.122, 2.222)	0.009	1.042 (0.472, 2.303)	0.918	1.851 (1.428, 2.398)	< 0.001	1.466 (0.857, 2.510)	0.163	1.476 (1.115, 1.954)	0.007
AG (T1)^a^												
≤ 22.3	Ref		Ref		Ref		Ref		Ref		1	
> 22.3	2.003 (1.317, 3.046)	0.001	1.213 (0.760, 1.934)	0.418	2.058 (0.833, 5.084)	0.118	1.192 (0.858, 1.658)	0.296	2.547 (1.178, 5.747)	0.024	1.293 (0.922, 1.812)	0.136
∆AG												
≤ 0	Ref		Ref		Ref		Ref		Ref		Ref	
> 0	0.755 (0.514, 1.109)	0.162	0.713 (0.502, 1.012)	0.058	0.603 (0.308, 1.196)	0.148	0.766 (0.580, 1.014)	0.062	0.582 (0.347, 0.978)	0.041	0.798 (0.599, 1.062)	0.122

*AG* anion gap, *ECI* Elixhauser comorbidity index, *eGFR* estimated glomerular filtration rate

Multivariate model adjusted for age, respiratory rate, heart rate, SBP, DBP, respiratory failure, SPO2, PLT, RDW, TBIL, eGFR, urine output, oxygen therapy, mechanical ventilation, vasopressor ECI, SAPSII, SOFA

^a^When analyzing AG (T1), multivariate model additionally adjusted for AG (T0) as a continuous variable

Sensitivity analysis was performed to explore the associations of AG (T0), AG (T1), and ∆AG with the 30-day mortality or 1-year mortality in patients using AG not adjusting for albumin (Table 5). The analysis showed very similar results to the main analysis shown in Table 2.

**Table 5:** Associations of AG (T0), AG (T1), and ∆AG with the 30-day mortality or 1-year mortality in patients when not adjusting for serum albumin concentration

	30-Day mortality				1-Year mortality			
	Univariate model		Multivariate model ^a^		Univariate model		Multivariate model^a^	
	HR (95% CI)	*P*	HR (95% CI)	*P*	HR (95% CI)	*P*	HR (95% CI)	*P*
AG (T0)								
≤ 18	Ref		Ref		Ref		Ref	
> 18	1.872 (1.420, 2.468)	< 0.001	1.931 (1.440, 2.590)	< 0.001	1.545 (1.241, 1.924)	< 0.001	1.774 (1.408, 2.237)	< 0.001
AG (T1)^b^								
≤ 19	Ref		Ref		Ref		Ref	
> 19	2.380 (1.768, 3.203)	< 0.001	2.011 (1.417, 2.853)	< 0.001	1.806 (1.413, 2.308)	< 0.001	1.564 (1.159, 2.064)	0.003
∆AG								
≤ 0	Ref		Ref		Ref		Ref	
> 0	0.581 (0.432, 0.780)	< 0.001	0.671 (0.491, 0.916)	0.012	0.678 (0.536, 0.857)	0.001	0.781 (0.610, 1.001)	0.051

*AG* anion gap

^a^Multivariate model adjusted for age, respiratory rate, heart rate, SBP, DBP, respiratory failure, SPO2, PLT, RDW, TBIL, eGFR, urine output, oxygen therapy, mechanical ventilation, vasopressor ECI, SAPSII, SOFA

^b^When analyzing AG (T1), multivariate model additionally adjusted for AG (T0) as a continuous variable

## Discussion

The current study evaluated the associations of AG (T0), AG (T1), and ∆AG with the 30-day mortality or 1-year mortality in critically ill patients receiving RRT. The results delineated that higher AG (T0) and AG (T1) while lower ∆AG were associated with higher risk of 30-day and 1-year mortality in these patients. The findings of this study might highlight the importance of monitoring AG and its changes at different time points for fluid management before and during RRT in critically ill patients requiring dialysis.

The serum AG is a calculated entity applied for assessing acid–base disorders and differential diagnosis of metabolic acidosis [18]. There was evidence indicated that anions that accumulating in patients on hemodialysis were associated with the lower ionized fraction of magnesium and calcium, which was an effective predictor for ionized magnesium and calcium in patients on hemodialysis [19]. Previously, Chen et al. revealed that the baseline AG ≥ 20 was associated with 1.63-fold increase in the risk of mortality of patients with congestive heart failure and chronic kidney disease [20]. In the study of Asahina et al., the association between AG and renal outcomes was evaluated in 1168 Japanese chronic kidney disease patients, which uncovered that high AG acidosis developed during the later stages of chronic kidney disease and the AG level changed with progression of chronic kidney disease [21]. Another study depicted that elevated serum AG in adults with moderate chronic kidney disease was associated with increased risk for progression to end-stage renal disease [22]. A high AG was reported to be an essential prognostic factor in patients with chronic kidney disease, because veverimer, a nonabsorbable hydrochloride-binding polymer, was validated to improve kidney function and decrease the AG [12]. In our study, we found that both higher AG before dialysis and higher AG 1-day after dialysis were correlated with increased risk of 30-day and 1-year mortality in critically ill patients undergoing RRT. Furthermore, ∆AG < 0, indicating that AG level 1-day after dialysis was lower than AG level before dialysis, was associated with decreased risk of 30-day and 1-year mortality in critically ill patients undergoing RRT. In previous studies, the change of AG was regarded as a novel marker of outcome in critically ill patients [23]. Lipnick et al. demonstrated that the changes between critical care initiation AG and prehospital admission AG was a predictor for mortality in patients with critical illness [24]. The mechanism might due to that the uremic acids such as indoxyl sulfate, p-cresyl sulfate, and trimethylamine N-oxide can cause kidney injury-induced renal fibrosis, which further increase the risk of mortality [25, 26]. This reminded the clinicians to concern on AG levels before and 1-day after dialysis, as well as their differences in critically ill patients undergoing RRT, and timely interventions should be provided to control their AG level.

Increased AG change was identified to be a risk factor for early mortality after starting hemodialysis in the elderly, and patients with high AG presented more advanced chronic kidney disease and more severe hyperphosphatemia [11]. Herein, subgroup analysis showed that increased AG level before dialysis was associated with elevated risk in 30-day mortality and 1-year mortality in those aged ≥ 65 years. High AG and increased AG level can result in irreparable damage in elderly patients including increased muscle protein catabolism with muscle wasting, reduced albumin synthesis with malnutrition, promotion of systemic inflammation, increased bone resorption [27]. Another study delineated that patients with high AG presented mobility impairments at the time of starting hemodialysis and preservation of mobility was one of the most important factors to improve the short-term prognosis after starting dialysis in elderly patients with end-stage renal disease [28]. The eGFR is a biomarker for the evaluation of chronic kidney disease, which can reflect preoperative renal function and is unitized for guiding the postoperative fluid management to prevent postoperative acute kidney injury in surgical populations [29]. In the current study, high AG level before dialysis was associated with high risk of 30-day and 1-year mortality in critically ill patients undergoing RRT who had eGFR ≥ 15; High AG level 1-day after dialysis and ∆AG were correlated with the risk of 30-day mortality in critically ill patients undergoing RRT who had eGFR < 15. For patients with eGFR < 15, clinicians should be pay special attention on AG level after dialysis and the changes of AG level before and after dialysis. ECI measures the clinical conditions that exist before hospital admission, which might be related to the mortality and resource use in the hospital [30]. Patients with high ECI score should be careful with AG levels and changes of AG level before and after dialysis.

The strength of our study was that we analyzed AG level at different time points to clear its changes and association with risk of 30-day and 1-year mortality in critically ill patients undergoing RRT. The finding might help the clinicians better manage these patients and make appropriate treatments for them. Several limitations existed in the current study. Firstly, this was a retrospective study, which might cause bias in the data. Secondly, the reason of patients receiving RRT was not clear, and we conducted the analysis in subgroup based on eGRF to reduce the influence of this limitation. Thirdly, due to the limitations of the MIMIC-III database, the data on the treatments after discharge as well as the detection methods of AG related indexes including sodium, potassium, chloride, bicarbonate and albumin could not be obtained, which might influence the results in our study. In the future, more studies were required to verify the findings of this study.

## Conclusion

The present study evaluated the associations of AG (T0), AG (T1), and ∆AG with the 30-day mortality or 1-year mortality in critically ill patients receiving RRT. We found AG before and after dialysis as well as the changes of AG were important prognostic biomarkers in critically ill patients receiving RRT. Dynamic monitoring of the changes helps improve bedside management of clinicians for patients. The results might provide a reference for the clinicians to continuously monitor AG levels and its changes at different time points for fluid management before and during RRT in critically ill patients requiring dialysis.

### Supplementary Information

Below is the link to the electronic supplementary material.

Supplementary Figure 1 The cumulative survival probability of 30-day mortality among participants as stratified by AG (T0) levels (PDF 76 KB)

Supplementary Figure 2 The cumulative survival probability of 1-year mortality among participants as stratified by AG (T0) levels (PDF 74 KB)

Supplementary Figure 3 The cumulative survival probability of 30-day mortality among participants as stratified by AG (T1) levels (PDF 75 KB)

Supplementary Figure 4 The cumulative survival probability of 1-year mortality among participants as stratified by AG (T1) levels (PDF 73 KB)

Supplementary Figure 5 The cumulative survival probability of 30-day mortality among participants as stratified by ∆AG levels (PDF 75 KB)

Supplementary Figure 6 The cumulative survival probability of 1-year mortality among participants as stratified by ∆AG levels (PDF 72 KB)

## Data Availability

The datasets used and/or analyzed during the current study are available from the MIMIC III database, https://mimic.physionet.org/iii/.
