# Supplementary figures and images for: Associations of continuous anionic gap detection with the mortality in critically ill patients receiving renal replacement therapy
Source: Int Urol Nephrol. 2023 Apr 7;55(11):2967–80. doi: 10.1007/s11255-023-03583-4 (PMC10560184; doi:10.1007/s11255-023-03583-4)

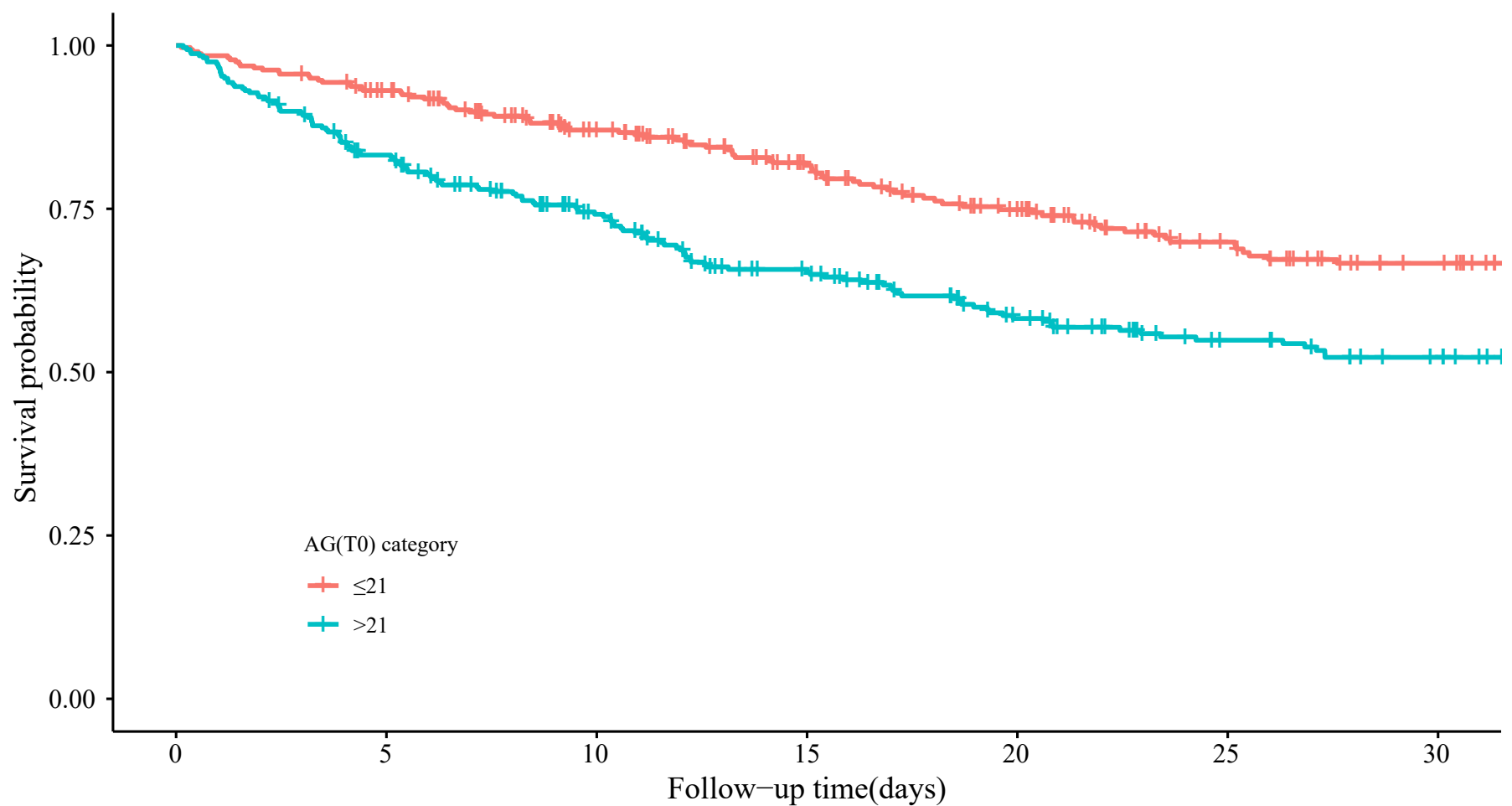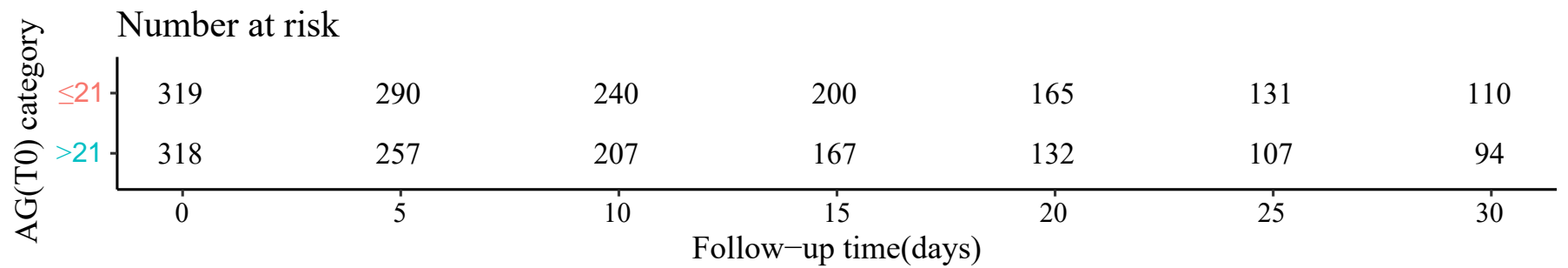

Supplement: Supplementary file 1 — Supplementary Figure 1 The cumulative survival probability of 30-day mortality among participants as stratified by AG (T0) levels (PDF 76 KB) [file 11255_2023_3583_MOESM1_ESM.pdf]

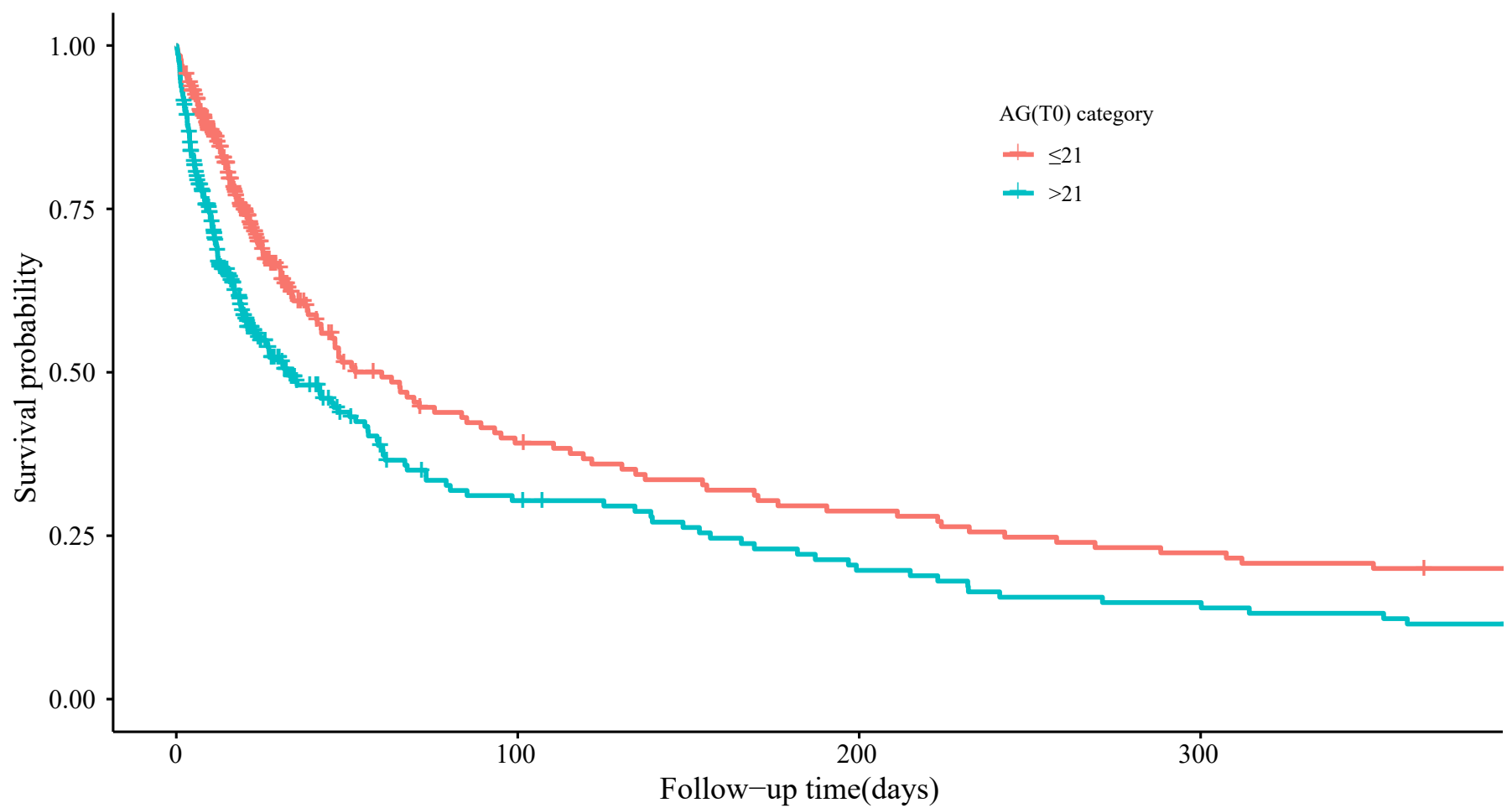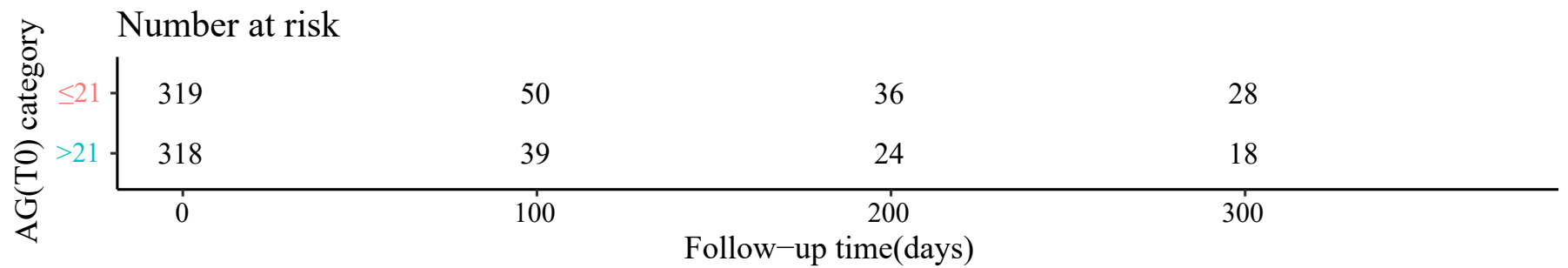

Supplement: Supplementary file 2 — Supplementary Figure 2 The cumulative survival probability of 1-year mortality among participants as stratified by AG (T0) levels (PDF 74 KB) [file 11255_2023_3583_MOESM2_ESM.pdf]

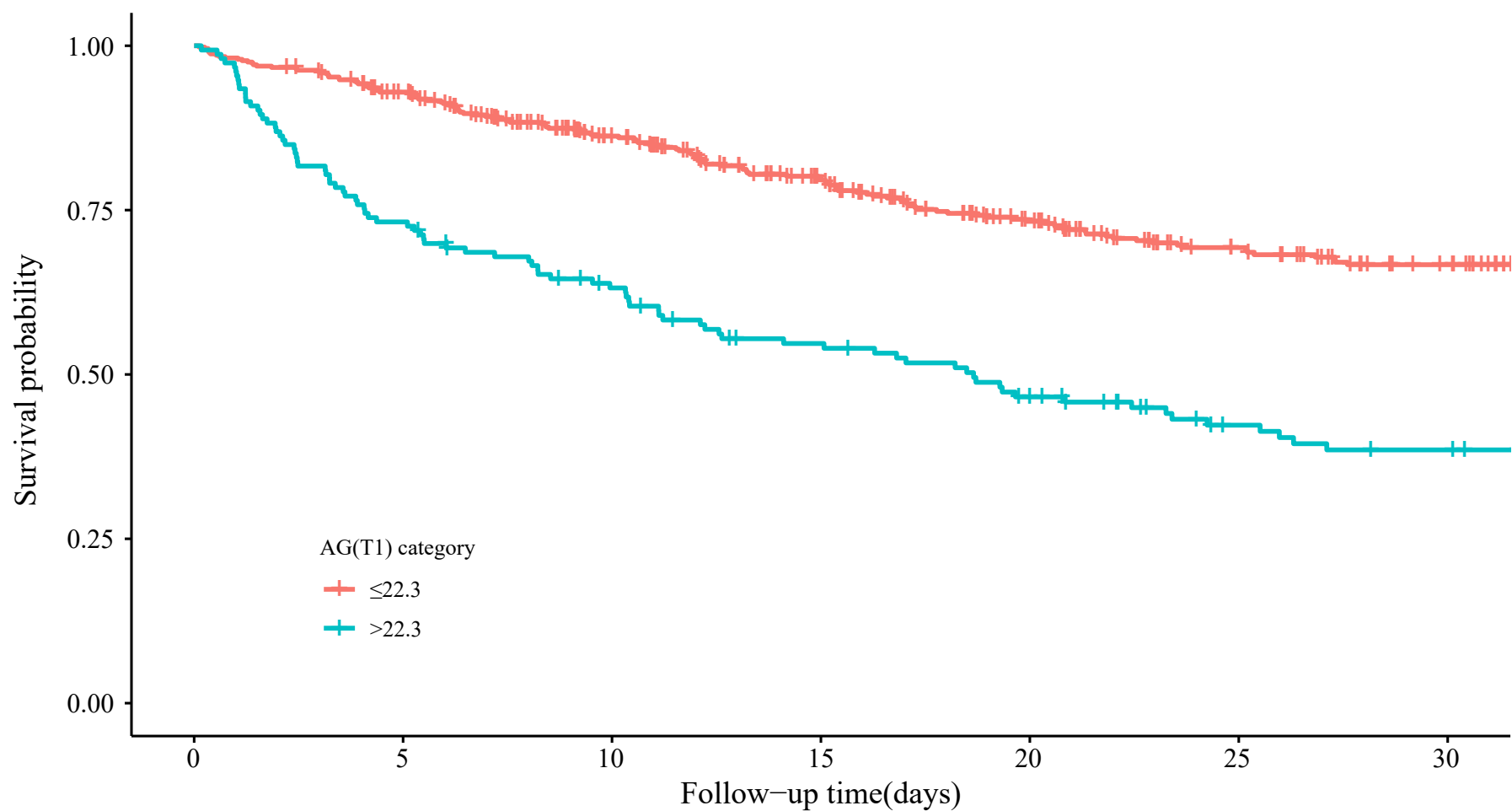

Number at risk

| AG(T1) category | 0   | 5   | 10  | 15  | 20  | 25  | 30  |
|-----------------|-----|-----|-----|-----|-----|-----|-----|
| $\leq 22.3$     | 484 | 435 | 356 | 292 | 236 | 193 | 164 |
| $> 22.3$        | 153 | 112 | 91  | 75  | 61  | 45  | 40  |

Follow-up time(days)

Supplement: Supplementary file 3 — Supplementary Figure 3 The cumulative survival probability of 30-day mortality among participants as stratified by AG (T1) levels (PDF 75 KB) [file 11255_2023_3583_MOESM3_ESM.pdf]

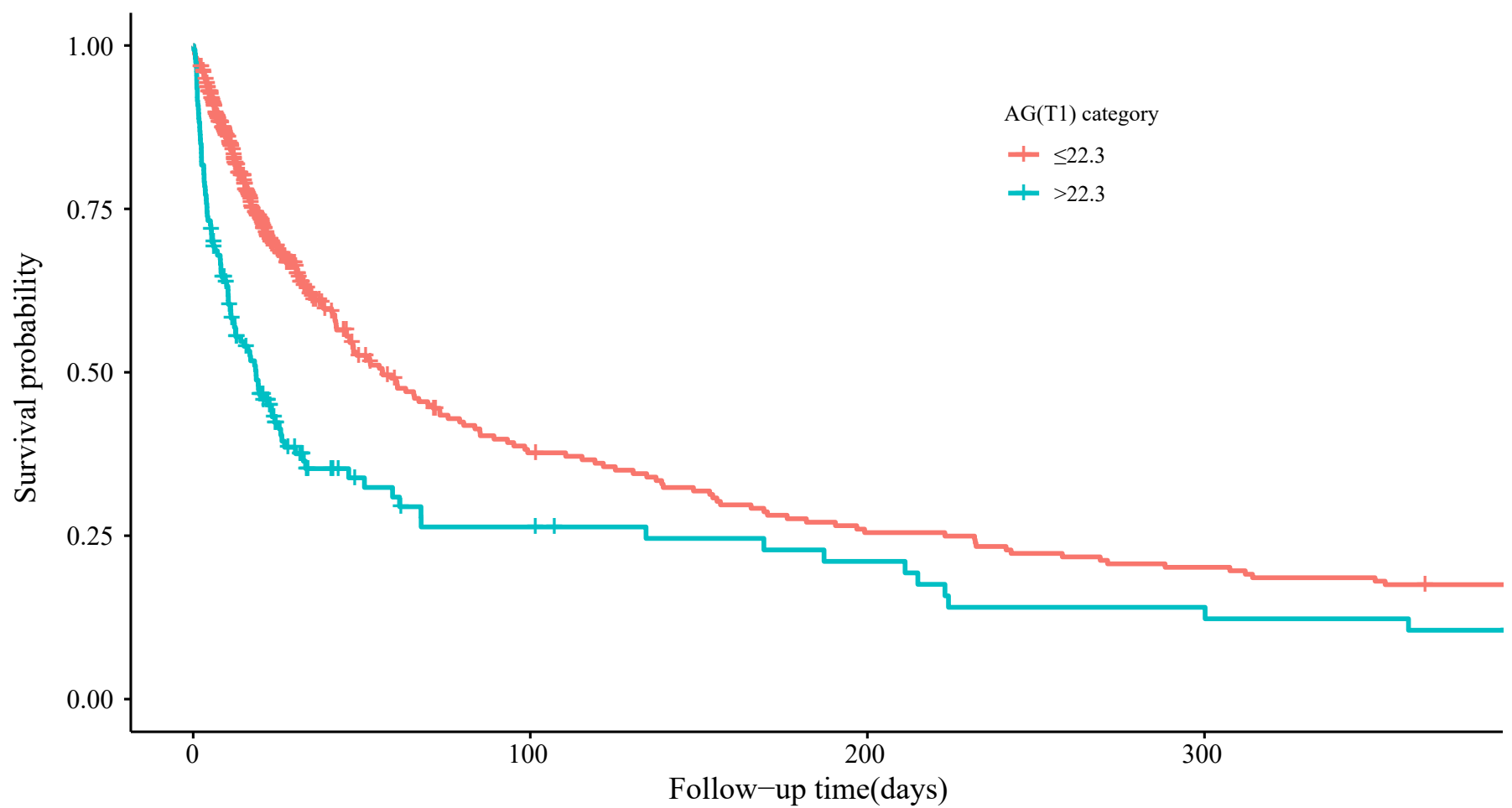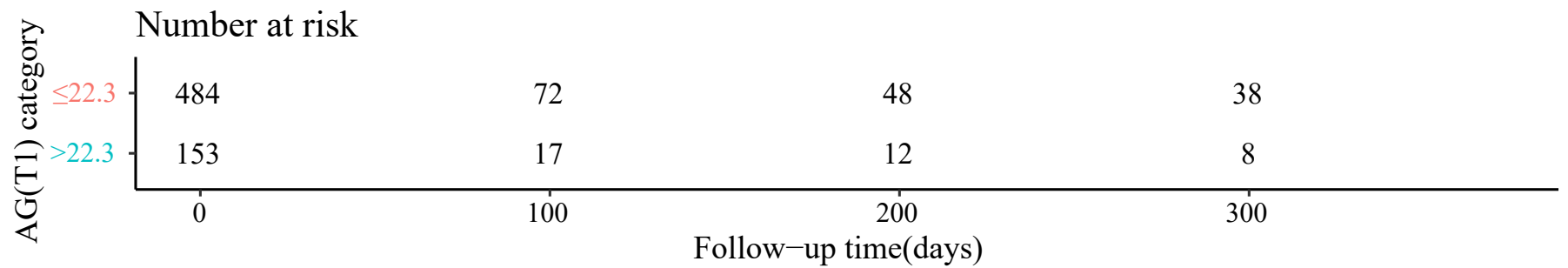

Supplement: Supplementary file 4 — Supplementary Figure 4 The cumulative survival probability of 1-year mortality among participants as stratified by AG (T1) levels (PDF 73 KB) [file 11255_2023_3583_MOESM4_ESM.pdf]

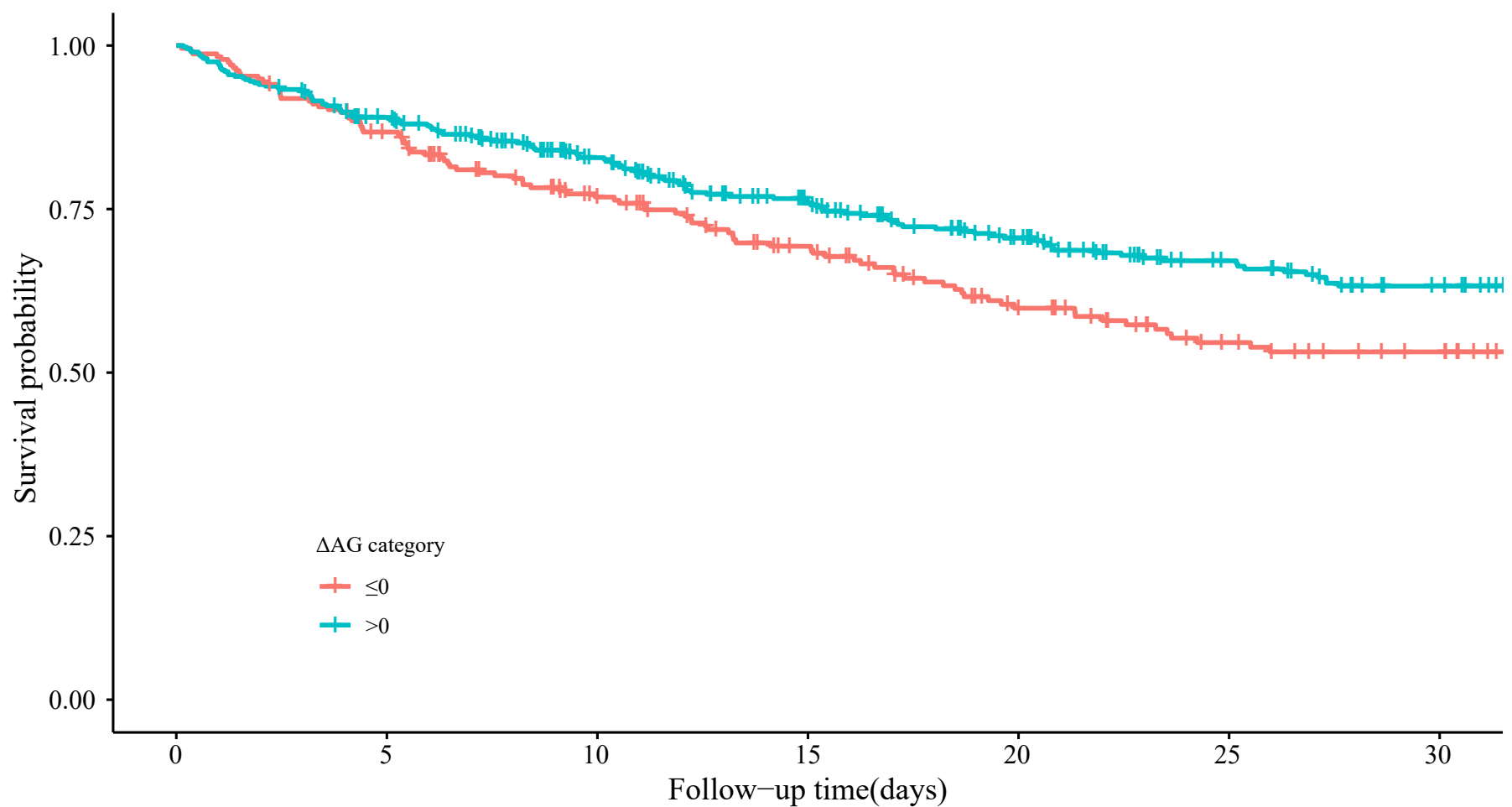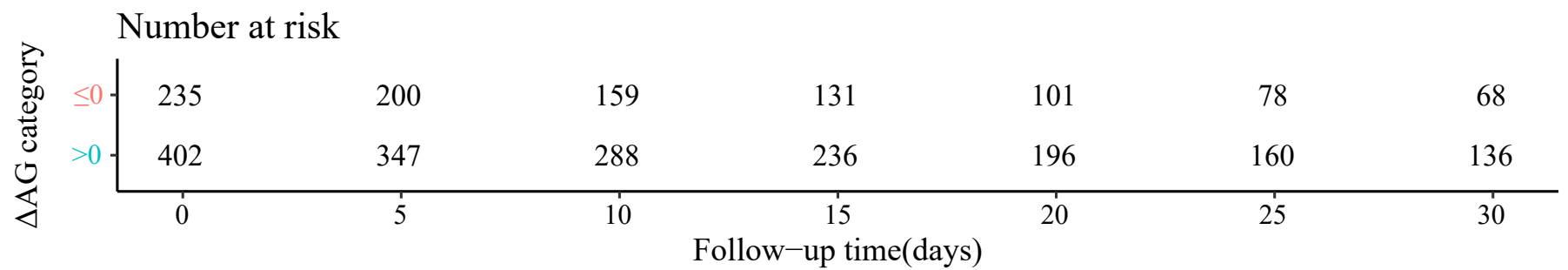

Supplement: Supplementary file 5 — Supplementary Figure 5 The cumulative survival probability of 30-day mortality among participants as stratified by ∆AG levels (PDF 75 KB) [file 11255_2023_3583_MOESM5_ESM.pdf]

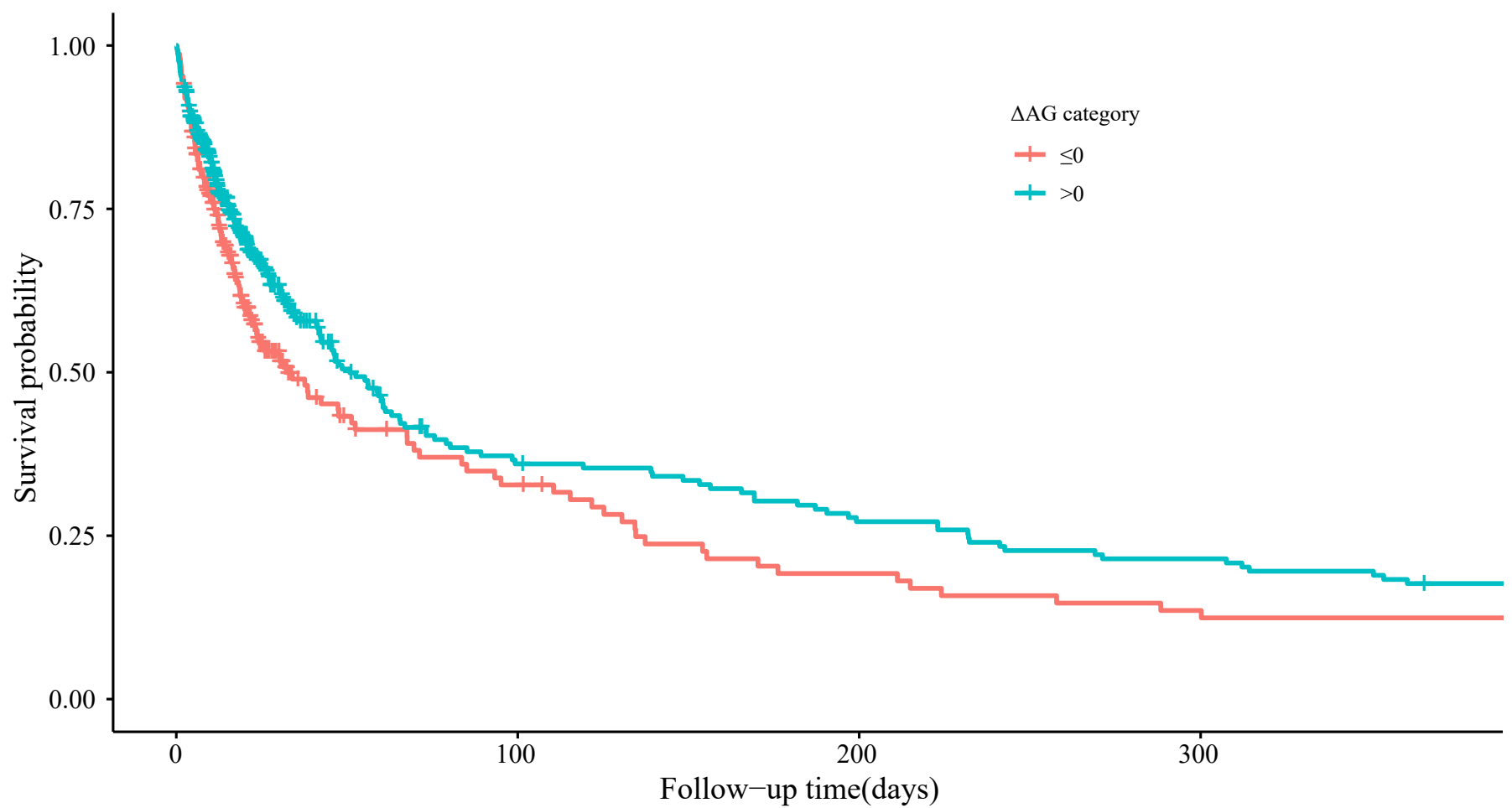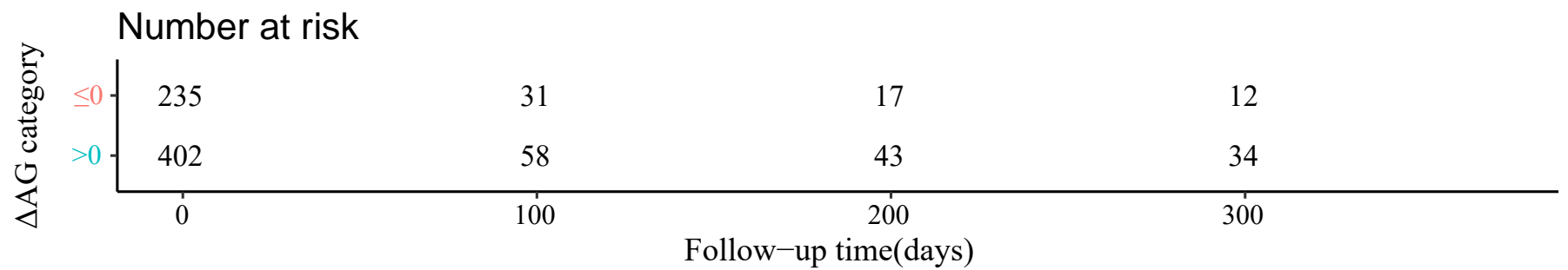

Supplement: Supplementary file 6 — Supplementary Figure 6 The cumulative survival probability of 1-year mortality among participants as stratified by ∆AG levels (PDF 72 KB) [file 11255_2023_3583_MOESM6_ESM.pdf]
